# Autoantigen Discovery in the Hair Loss Disorder, Alopecia Areata: Implication of Post-Translational Modifications

**DOI:** 10.3389/fimmu.2022.890027

**Published:** 2022-06-03

**Authors:** Shahnawaz D. Jadeja, Desmond J. Tobin

**Affiliations:** ^1^ The Charles Institute of Dermatology, School of Medicine, University College Dublin, Dublin, Ireland; ^2^ The Conway Institute of Biomolecular and Biomedical Research, University College Dublin, Dublin, Ireland

**Keywords:** anagen hair follicle, autoimmunity, immune privilege, post-translational modification (PTM), citrullination, deamidation, autoantigen, trichohyalin (TCHH)

## Abstract

Alopecia areata (AA) is a chronic, multifactorial, polygenic, and heterogeneous disorder affecting growing hair follicles in susceptible individuals, which results in a non-scarring and reversible hair loss with a highly unpredictable course. Despite very considerable research effort, the nature of the precipitating factor(s) responsible for initiating AA in any given hair follicle remains unclear, due largely to significant gaps in our knowledge of the precise sequence of the etiopathogenic events in this dermatosis. However, disease-related changes in the immune-competence of the lower growing hair follicle, together with an active immune response (humoral and cellular) to hair follicle-associated antigens, are key associated phenomena. Confirmation of the hair follicle antigen(s) implicated in AA disease onset has remained stubbornly elusive. While it may be considered somewhat philosophical by some, it is also unclear whether immune-mediated hair loss in AA results from a) an ectopic (i.e., in an abnormal location) immune response to native (unmodified) self-antigens expressed by the healthy hair follicle, b) a normal immune response against modified self-antigens (or neoantigens), or c) a normal immune response against self-antigens (modified/non-modified) that were not previously visible to the immune system (because they were conformationally-hidden or sequestered) but become exposed and presentable in an MHC-I/-II molecule-restricted manner. While some candidate hair follicle antigen target(s) in AA are beginning to emerge, with a potential role for trichohyalin, it is not yet clear whether this represents the *initial* and *immunodominant* antigenic focus in AA or is simply one of an expanding repertoire of exposed hair follicle tissue damage-associated antigens that are secondary to the disease. Confirmation of autoantigen identity is essential for our understanding of AA etiopathogenesis, and consequently for developing a more informed therapeutic strategy. Major strides have been made in autoantigen discovery in other autoimmune conditions. In particular, some of these conditions may provide insights into how post-translational modifications (e.g., citrullination, deamidation, etc.) of hair follicle-restricted proteins may increase their antigenicity and so help drive the anti-hair follicle immune attack in AA.

## 1 Introduction

The hair follicle (HF) is the most prominent appendage of the skin and is a uniquely mammalian trait (1). Its secreted product, the hair fiber, is of considerable psycho-social importance for humans, rendering disorders of the HF highly impactful on our quality of life (2). Alopecia areata (AA), is a relatively common (lifetime risk of ~2%) immune-mediated disorder of HF growth, which can affect males and females of all ages and geographic ancestries (3–5). The diagnosis of AA is usually straightforward. It most commonly presents with patchy areas of hair loss on the scalp (so-called patchy AA) but can progress to loss of all scalp hair (A. totalis) and body hair (A. universalis).

AA is one of the most enigmatic of the common dermatoses, in large part due to its exceedingly unpredictable course, heterogeneous presentation, in the context of the unusual mosaic nature of human scalp hair growth where much autonomy is invested in each individual HF. Despite intimations to the contrary, the etiology of AA remains unknown. AA is associated, however, with several immune abnormalities, some non-selective while others appear to be more specific and point to an immune abnormality that targets a component(s) of the HF, especially when in the anagen phase of the hair growth cycle. Still, AA may also have features of systemic disease, as is characterized by frequent involvement of other tissues that so far include nails, eyes, and heart (6–9). AA is most readily seen on areas of the body with the highest proportion of HF in the growth phase of the hair cycle (called anagen) e.g., scalp and beard. However, it may appear on any hair-bearing sites (10, 11). AA has also been reported in several non-human mammalian species e.g., horses, dogs, inbred mice, rats, rhesus monkeys, cows, etc. (12–19). Treatment of AA is difficult; there is no FDA-approved drug, and there is currently no cure. Despite this, a broad range of treatment modalities can be used, including topical, systemic, and intralesional corticosteroids; topical immunotherapy; topical minoxidil; anthralin; and Psoralen plus Ultraviolet A (PUVA), and more recently JAK inhibitors (20, 21).

In this review we re-examine what is known about the antigenicity of HF proteins, potential HF autoantigens in AA, and whether post-translational modifications of the presumptive HF autoantigen trichohyalin, may render this crucial HF protein susceptible to triggering an inappropriate immune response to the HF in AA-susceptible patients. Specifically, we examine how two post-translational modifications (citrullination and deamidation) that can alter protein antigenicity, may be particularly relevant in the *induction* of AA.

## 2 Etiopathogenesis of Alopecia Areata

Despite a very concerted research effort over the last 30 years, the etiology of AA remains elusive. What we call AA may even represent more than one disease entity. However, for simplicity, we can describe AA as multifactorial with a strong genetic predisposition in affected kinships (22, 23). There is a striking acquisition of ectopic immune-competence (most easily represented by upregulated MHC-I expression) in the cycling part of the growing HF (24), as well as other features of immunological dysregulation (25, 26). Dysregulated redox balance (27, 28), and microbial dysregulation (29, 30) may also feature in AA pathogenesis. While these and numerous other factors have been reported in AA over the decades (31), the classical features of active disease include the brisk infiltration of immune cells in a classic ‘Swarm of Bees’-like pattern (32), and the production of autoantibodies to HF-associated proteins (33–35). Recently, in an interesting transcriptomics study, Borcherding et al., observed clonal expansions of both CD4^+^ and CD8^+^ T cells, with shared clonotypes across varied transcriptional states in murine and human AA, suggesting autoantigen-dependent cellular autoimmune response in AA (36).

The curiously low immune-competence of the lower anagen HF is characterized by a range of devices, including the maintenance of low expression of classical MHC-I molecules in the HF epithelial components. These are described in considerable detail elsewhere (22, 24) and so will only be briefly alluded to here. This unusual low immune-competence of the epithelium in the growing HF is likely to be essential for the life-long cyclical (i.e., regenerative) nature of hair growth. Significant evolutionary selective pressure likely developed a range of devices to prevent the inadvertent and catastrophic immune-mediated damage to the mammal’s HF-dense coat in the wild (37). Thus, the growing HF, together with a small number of other sites (anterior chamber of the eye, placenta/fetus, testes, and central nervous system excluding brain) are able to ‘tolerate’ the production of antigens (self, non-self, modified, neoantigens, etc.) without eliciting an inflammatory immune response and so protect this vital skin appendage from direct or collateral damage.

Antigens in these appendages interact with T cells in an unusual way, including by inducing a form of tolerance to normally rejected stimuli (38). This is likely to be especially relevant in haired skin, where HF ostia provide for millions of potential ports-of-entry across our skin surface to ensure continuous connection between the skin organ and the outside world. However, to do this successfully, the skin must balance risks from the entry of microbes and other potential aggressins. Indeed, it is far from sterile under the epidermis of the skin, with the highest expression of prokaryotic 16S rRNA detectable at or close to the depth of the scalp anagen hair bulb (i.e. ~3 mm deep) (39).

AA is essentially a hair growth cycle disorder; as it presents with the greatest vulnerability to immune-mediated attack during early anagen, at a time coincident with the re-establishment of the HF fiber and melanin production systems (40). The HF is the only continually-cycling tissue throughout the entire lifespan of the mammal (41), and is an impressive example of a physiological deconstruction of a multicellular tissue (via programmed death called apoptosis), followed by its reconstruction (via massive cell proliferation, differentiation, and maturation) to facilitate a new generation of hair fiber growth. After initiation at the start of anagen (operationally divided into anagen I-V), active hair fiber production continues during anagen VI for a long time, typically 3-5 years on the human scalp, but in some people even longer, before coming to an end again with the involution of up to 70% of the growing hair follicle during a phase called catagen (42, 43). The catagen HF then transitions to a phase of relative dormancy (called telogen), around which time the hair fiber can be ejected from the human HF *via* a process called exogen (44). Then, the HF re-enters a new hair fiber-producing anagen phase, or in the aging mammal can remain empty as a so-called ‘kenogen’ HF for some further time (45). Thus, as the greatest vulnerability to immune-mediated attack in AA occurs only during early anagen, we must concentrate more attention on what is going on in the HF at this particular time. Moreover, if one attempts to search only for the footprints in the wake of key immune-mediated events in the AA immune attack, it is likely to be much too late.

## 3 Discovery of AA-Related Hair Follicle Autoantigens – An Elusive Holy Grail

In this game of dominoes, the immune cascade of successively falling tiles has already begun when HF tissue damage or immune cell infiltration has already become evident (i.e., the thief has been and gone). Moreover, to turn off this immune assault in early AA lesions or to stop/protect it from starting in the first place, one needs to identify the mover(s) of the first falling domino as well as identifying the nature of the first domino itself. In our view, this is likely to center on ‘flag-waving’ change(s) in the targeted HF i.e., the antigenic flags that first attract the attention of a misguided immune response. Given that this HF disorder commonly targets people with a (genetic) predisposition/susceptibility to inflammation and autoimmunity (e.g., atopy, Hashimoto’s thyroiditis, pernicious anemia, celiac disease, etc.), a preferred mode of the (auto)inflammatory response to the HF may already be established in these patients *via* an already altered immune-activation threshold (22, 46). Thus, we need to determine the cellular and tissue events that pre-date induction of ectopic immune-competence in the AA-susceptible lower HF. The latter is prevented under normal circumstances by an active or induced state of immune privilege or tolerance unique to key AA-targeted epithelial components of the HF (2, 47). Early work by Bystryn and others reported the presence of abnormal immune deposits in the lower HF in AA patients, which suggested this region of the HF to be a prime target of pathogenic autoinflammatory attention (40, 48, 49). This was further confirmed with the observation of circulating antibodies in the sera of AA patients that bound to proteins extracted from scalp anagen HF (33), and later to distinct compartments of the anagen HF itself (34). Patients with autoimmune polyendocrine syndrome type I (APS I) - a condition where up to 37% can have AA (50) - are also observed to have high titre autoantibodies directed against the anagen bulb matrix, hair fiber cuticle, and cortex keratinocytes and also against melanocyte nuclear antigens (51). Thus, it is evident from several studies that anti-HF autoantibodies, in addition to cellular drivers, are associated with AA pathogenesis, even if not directly as effector molecules. Still, most attention has been directed toward the cellular arm of the immune response to HF in AA, leaving humoral factors relatively ignored to date.

Histopathological observations suggest that the hair growth cycle is disrupted in AA, due to the premature involution of early anagen (anagen III-VI) HF into catagen (52, 53). This is key, as AA does not present with an inflammatory response to the overlying epidermis or even the upper non-cycling parts of the HF (infundibulum and isthmus) - despite that, cells of the same histological type reside there i.e., keratinocytes, melanocytes, etc. Thus, there must be something *unique* happening in the cycling part of the AA-susceptible HF during early/earliest anagen, which induces, exposes or in some other manner activates an auto-inflammatory process. As a result, the soon-to-be targeted anagen hair bulb is forced to leave its protective immune silence and move above the radar with often catastrophic consequences.

While the involvement of cellular (intra-/perifollicular immune cell infiltrates) and humoral (anti-HF autoantibodies) immunity is well-established in AA, much less progress has been made to identify the HF-associated autoantigen(s) that induces pivotal stages in the pathogenesis of this disorder (25, 31, 49). Recently, some have even proposed that the phenotype of AA may be derived from alternatively autoimmune and non-autoimmune pathomechanisms (54), with the latter mode being largely independent of autoantigens. Still, a small group of AA researchers has persisted against all (including funding) odds with their attempts to discover relevant autoantigens in AA. They do this, not only to determine whether AA can be definitively classified as truly ‘auto-immune’, but also to explore whether tolerization to a self-HF specific antigen(s) could shut-off the immune-mediated attack on the anagen HF and so treat (or even cure) this frustrating skin condition.

To date, a few potential autoantigens in AA have been suggested (**Table 1**) and these are mostly derived from HF keratinocytes and HF melanocytes. The most prominent of these, *Trichohyalin* (TCHH) is located mostly in the HF inner root sheath (IRS) and importantly is only synthesized during the anagen stage of hair growth. Support for the involvement of TCHH comes from a variety of sources. Early work has shown the common expression of circulating IgG autoantibodies to TCHH in human (55) and animal (16, 56) patients with AA hair loss, and later work showed T cell reactivities to this protein in AA patients (57) (see below for a detailed discussion of TCHH as a presumptive autoantigen in AA). However, from these data, it is not yet clear which autoantigen(s)/epitope(s) is the *first* target of the inappropriate attention of the AA immune-system. We need to distinguish between targets of the initial stage of AA onset versus the antigens/epitopes that emerge during antigenic drift and epitope spread as AA disease progresses and/or in disease of long-standing (49, 55, 57, 60, 63, 64). Thus, discovery of the key HF-associated autoantigens that are truly responsible for triggering the initial immune-mediated cascade against the anagen HF in AA may help us to better understand disease onset and progression. Furthermore, identifying key autoantigens could possibly pave the way for developing novel therapeutic approaches.

**Table 1:** List of putative autoantigens identified in Alopecia areata.

Sr. No.	Autoantigen	References
1.	Trichohyalin (TCHH)	(16, 55–58)
2.	Keratin 16 (K16)	(55)
3.	Fibroblast growth factor receptor 3 (FGFR3)	(59)
4.	Glycoprotein-100 (gp100)	(60)
5.	Melanoma antigen recognized by T cells 1 (MART1)	(60)
6.	Dopachrome Tautomerase (DCT)/Tyrosinase-related protein 2 (TYRP2)	(61)
7.	Tyrosinase (TYR)	(61)
8.	Tyrosine hydroxylase (TH)	(62)

Previously, we observed that hair bulb keratinocytes and hair bulb melanocytes were primarily affected in acute AA (34, 53, 58), suggesting some involvement of (precursor) keratinocyte- or melanocyte-associated antigens, which may become visible to the immune system as a result of either upregulation of immune-competence in targeted HF (e.g., *via* ectopic MHC-I/-II molecule expression) with or without the help of local antigen-presenting cells.

### 3.1 Melanocyte-Related Hair Follicle Autoantigens in AA

AA is associated with several pigmentary anomalies, including the preferential targeting of pigmented HF, the (relative) sparing of white HF, as well as regrowth of initially-depigmented hair (65). Ultrastructural examination of AA-affected anagen HF revealed hair bulb melanocytes with aberrant melanogenesis and undergoing apoptosis-like degeneration (40). Given that melanogenesis and melanosome transfer to cortical hair bulb keratinocytes are strictly coupled to the anagen III-IV stage (66, 67), melanocyte-related proteins have long been viewed as potential early targets in AA (60, 64, 68). Moreover, AA onset was observed in C57/BL6 mice following successful immunotherapy for melanoma, suggesting the presence of pigment cell-associated antigens in AA pathogenesis (68). Others have suggested melanocyte-derived autoantigens in AA (**Table 1**), including epitopes of the melanosomal protein gp100 and the Melanoma antigen recognized by T cells-1 (MART1) (60). Recently, Hashimoto and colleagues reported autoantibodies and CD8^+^ T cells that reacted with tyrosinase (TYR) and dopachrome tautomerase (also known as tyrosinase-related protein-2) in acute and chronic AA-like disease in a C3H/HeJ mouse model (61). While melanocyte-derived autoantigens may be a potential target in AA, we should not ignore the observation that melanocytes in the overlying immune-competent (MHC-I positive) epidermis are spared in AA, unlike their fate in vitiligo (31, 69). This in part could be explained by the observation that melanocyte populations are morphologically and antigenically distinct in these two cutaneous compartments (70). Thus, HF melanocyte-derived antigenic targets could be among the motifs altered in AA, due to local biochemical modifications of epitopes that are usually invisible or sequestered in the context of anagen-associated immune privilege in this part of the HF. However, as AA can still affect non-pigmented (gray/white) HFs (52), the immune response to HF melanocyte antigens may be a secondary (non-pathogenic) immune target e.g., resulting from antigenic drift after an initial encounter with a different ‘primary target’. The latter therefore more likely involves the super-active keratinocyte population undergoing enormous proliferation and exquisitely complex multilineage differentiation (71) to make the HF (e.g., IRS and ORS) and its fiber (e.g., cuticle, cortex, medulla).

### 3.2 Keratinocyte-Related Hair Follicle Antigens in AA

The most common structures targeted by autoantibodies in AA appear to be located in the HF supra-Auber’s hair bulb matrix, IRS and ORS (34, 58). This suggests that a significant element of the HF-specific immune response in AA is directed to keratinocytes that are still proliferating (i.e., undifferentiated), undergoing sub-lineage commitment, and early stages of differentiation in the anagen hair bulb. We have previously reported that 44/46 kDa type-I keratins, whose expression is restricted to the HF, are specifically targeted by circulating serum IgG in AA patients, and indeed also by sera of the C3H/HeJ mouse model of AA (70, 72). Despite some inter-individual AA patient heterogeneity, most human AA serum IgG antibodies target HF antigens of 40-60 kDa and ~220-250 kDa, and with a similar pattern in other mammals, including equine and canine AA counterparts (16, 33, 56). We previously assessed immunoprecipitated AA-reactive HF-specific autoantigens using AA patients sera by western blotting (70) and by mass spectrometric analysis. Using both techniques we identified the keratin intermediate filament-associated TCHH protein as a potential immunodominant autoantigen (55). The latter study also reported auto-reactivity to keratin 16 (K16) protein expressed in the ORS. In another study, Erb et al. observed that K71 (expressed in IRS), and K31 (expressed in lower hair cortex) could activate a T cell response in the C3H/HeJ mice model of AA. Importantly, these researchers observed that vaccination of these mice with soluble K71 or K31 peptides significantly halted AA progression (73).

#### 3.2.1 Trichohyalin - An Attractive Candidate Autoantigen in AA

Of all the putative HF autoantigens identified to date, our identification of TCHH as a putative immuno-dominant AA autoantigen is pathophysiologically highly relevant in our view. First, TCHH is a key HF structural protein - providing crucial mechanical strength by integrating with keratin intermediate filaments (KIF). Second, TCHH is expressed mostly in the IRS - an anagen-specific component of the HF (74). Indeed, intriguingly, TCHH is one of the earliest (if not the earliest) readily-detectable differentiation-associated markers in the new anagen HF (41). TCHH protein expression becomes apparent in the most proximal and importantly, peripheral areas of the anagen hair bulb - a location where the hair bulb still lacks obvious structural differentiation into either IRS or pre-cortex, and before the ORS is evident. Thus, TCHH-expressing pre-IRS cells distribute (counter-intuitively) to the *outside* of the HF epithelium at this stage in anagen development. These differentiating IRS keratinocytes are in very close proximity to the highly-vascularized and MHC-I positive perifollicular connective tissue sheath, wherein a wide diversity of (sentinel) immune cells (75), including mast cells, macrophages, neutrophils, Langerhans/Dendritic cells, even CD4^+^ and CD8^+^ T lymphocytes reside, albeit in very low numbers. In that way, autoantigens or neo-antigens expressed by precursor and differentiating IRS cells in this part of the lower anagen hair bulb are well-positioned for direct/close interaction with both steady-state and activated (e.g., post micro-trauma, immunization/vaccination, etc.) immune system components. The MHC-I-inducing cytokine, Interferon-γ, may be released following activation of resident peri- and intra-bulbar T cells, macrophages as well as activated mast cells in the mesenchyme, thus emphasizing that a mild-to-moderate pro-inflammatory environment may be needed to *precede* the brisk lymphocytic infiltration that drives tissue damage and hair loss in AA.

**Figure 1:**
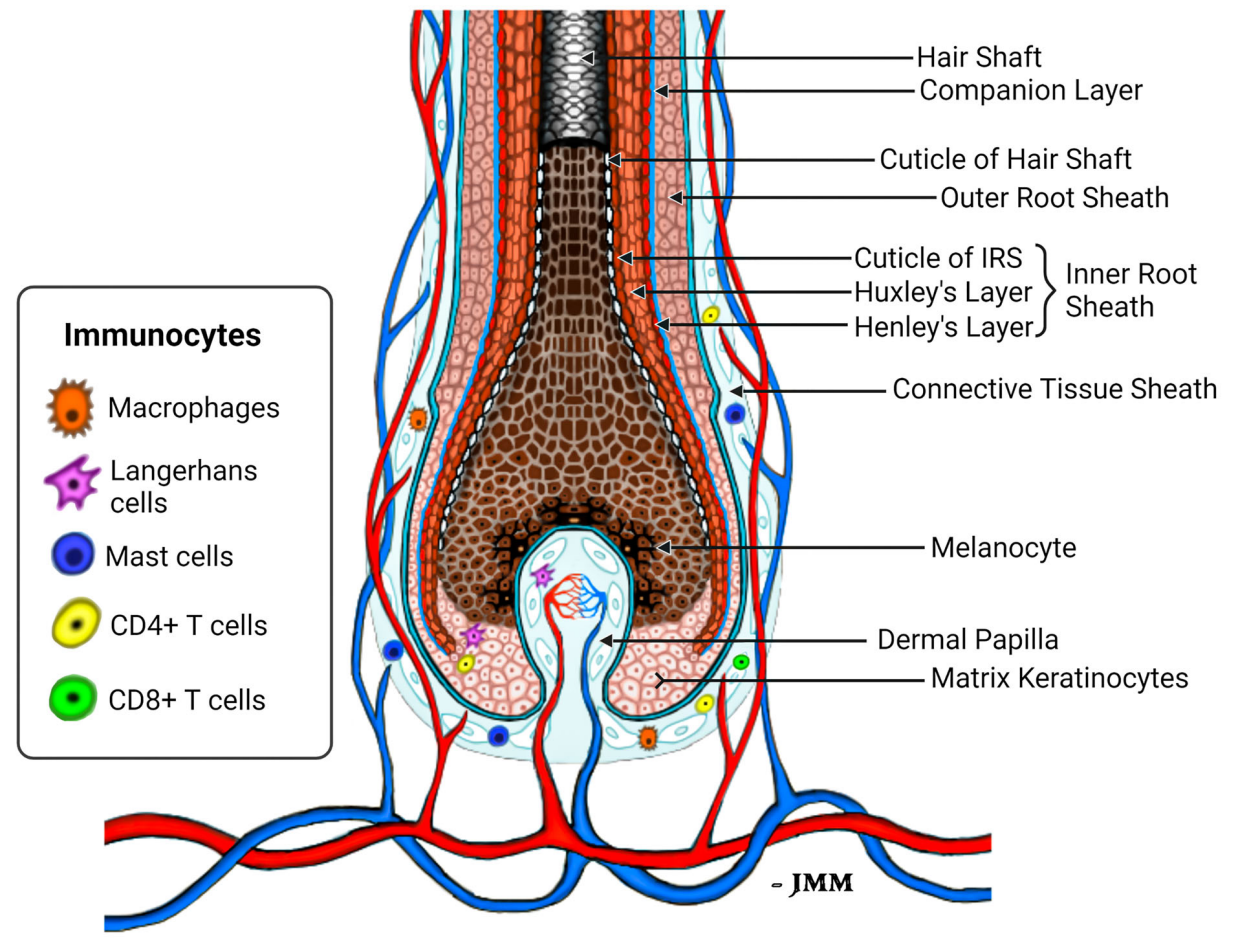
Schema of the healthy lower anagen scalp hair follicle. Different compartments of the HF bulb are shown, including the location of peri-follicular and intra-follicular vasculature and presence of immunocytes that normally reside in the HF connective tissue sheath and hair bulb epithelium (75).

**Figure 2:**
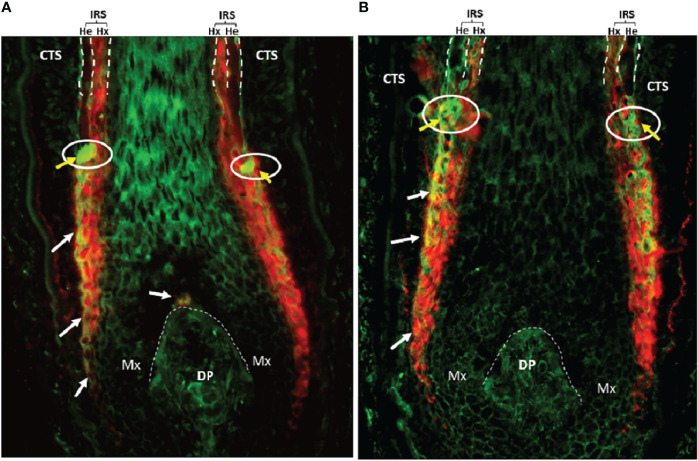
**(A)** AA patient #1 and **(B)** AA patient #2. AA serum IgG (green) and anti-TCHH (red) antibody colocalization in the anagen hair bulb (normal human scalp, 20x) as indicated by yellow/orange staining (white arrows). The white circle with yellow arrows highlights to AA serum IgG antibody binding to antigens located in the so-called ‘ring of fire’ redox-active zone of the HF (78). DP, Dermal Papilla; CTS, Connective Tissue Sheath; Mx, Matrix; IRS, Inner root sheath; He, Henle’s layer; Hx, Huxley’s layer.

Keratinocytes committed to the IRS lineage continue to develop in the supra-bulbar HF (**Figure 1**), where precursor IRS keratinocytes adopt one of three possible cell fates; most-externally as cells of the first-to-cornify Henle’s layer, then centrally as the Huxley’s layer, and finally as innermost IRS cuticle layer cells that imbricate with the cuticle cells of the hair fiber. All three layers of the IRS express TCHH as a very significant component protein (76, 77). Interference or disruption in IRS development, in this most transcriptionally- and translationally-sensitive and proliferative region of the early anagen hair bulb, would be expected to seriously impair the progression of hair growth (anagen). Instead such disruption is very likely to cause the HF to enter a premature involution or catagen state. Intriguingly, we have observed the presence of anti-TCHH IgG antibodies in AA patient sera that can target TCHH in those cells exhibiting very early IRS differentiation (55) (**Figure 2**). Indeed, AA antibody reactivity appears to preferentially target epitopes that show exquisite differentiation-associated timelines, as well as specific sub-IRS cell lineage expression profiles. Cells destined to become the externally-distributed Henle’s layer are most often targeted by AA anti-TCHH IgG antibodies.

TCHH protein expression first appears near the base of the developing anagen hair bulb, within non-membrane-bound granules, which gradually disperse and integrate with KIFs upon further maturation and differentiation of the pre-IRS keratinocyte (79, 80). As an insoluble α-helical and arginine-rich protein, TCHH undergoes sequential post-translational modifications (PTM) in order to become more soluble and more integratable with KIFs, in a process that characterizes progressive IRS maturation and differentiation. In brief, TCHH undergoes modifications in four main stages viz., i) accumulation into KIFs as insoluble droplets, ii) initial modifications by cytosolic peptidyl arginine deiminase (PADI) enzymes that catalyse the conversion of positively-charged arginine to neutrally-charged citrulline, resulting in conformational changes leading to its increased solubility and dispersal, iii) increasing solubility subsequently allows Transglutaminases (TGases) to catalyse deamidation of TCHH glutamine residues to glutamic acid, ensuing cross-linking of TCHH with KIFs in the IRS. Completion of TCHH processing results in a toughened (cornified) insoluble structure (81) that can help mould the pliable hair fiber within, as the latter grows and hardens. Thus, we consider that TCHH is particularly prone to express candidate autoantigens in AA by a) being one of the earliest expressed proteins in early anagen (Anagen III) and b) by being a protein substrate for PADIs and TGases – for creation of citrullinated or deamidated amino acids can play very crucial roles in the induction of several autoimmune disorders (82).

However, the investigation of the role of the PTMs citrullination or deamidation in TCHH amino acids in AA is only at its very beginning and is a major focus of our laboratory. As is often the case in research science, much can be learned from similar paradigms in other auto-inflammatory disorders that have undergone much deeper exploration to date.

## 4 Autoantigens and Post-translational Modifications: Lessons From Other Autoimmune Diseases

Autoimmune responses represent dysregulation of effector and regulatory immune mechanisms, which generally develop through phases of initiation and propagation, and often show periods of remission and relapse (83). Post-translational modification (PTM) is one of several normal devices for expanding or diversifying the functional protein repertoire, which can produce an extraordinarily complex ‘proteoform’ (84). Also, any one single protein may undergo significant modification during cellular differentiation and maturation. This plasticity of peptide epitope form is likely to influence the creation of potential (self)-antigens. PTMs can occur in self-proteins during the normal maturation/differentiation processes of cells, but may be further altered in the presence of additional cell stressors, such as oxidative stress, inflammation, aging/senescence, etc. How these (self)-antigens are perceived by an individual’s immune system will depend on a range of host factors, not least their susceptibility to autoreactivity. Prominent examples of the latter include associations with particular MHC-I and -II alleles (22), and polymorphisms including in *PTPN22* (encoding a lymphoid-specific tyrosine phosphatase that is a master regulator of the immune response), and *AIRE* (encoding a transcription factor expressed in the thymic medulla and involved in the elimination of self-reactive T cells) (85)-. However, alone these may be insufficient for disease development. Previous studies have shown that negative selection of T cells specific for the PTM-variant of the same antigen requires transport of the peripheral antigen (e.g., TCHH here) to the thymus by professional APCs (86). However, those studies suggest that peripheral tolerance mechanisms and/or transport of peripheral self-antigens to the thymus cannot compensate for the lack of autochthonous (i.e., indigenous) thymic-induced central tolerance.

APCs enter the affected tissue, engulf cellular damage and potentially transport these proteins to the lymph node for presentation in the context of MHC-I/-II molecules to lymphocytes and subsequent amplification of the antigenic repertoire. Consequently, this leads to infiltration of autoreactive T cells and B cells into the host tissue where that may drive an auto-inflammatory or autoimmune reaction (87). Several autoinflammatory disorders exhibit striking post-translational modifications in tissue-restricted proteins that trigger autoimmune responses (**Table 2**). In that way, genes that encode candidate antigens do not necessarily need to be reflected in patient GWAS studies. Still, Petukhova et al., reported previously that patients with AA exhibited several overlapping genetic susceptibility loci with other autoimmune diseases, including most commonly rheumatoid arthritis (RA), type I diabetes (T1D), and celiac disease (CD) (22), suggesting that these disorders may provide insights into the etiopathogenesis of AA. However, in terms of AA autoantigen discovery, our knowledge lags far behind that of autoantigens in these more definitive autoimmune conditions.

**Table 2:** Post-translationally modified autoantigens reported in other autoimmune disorders.

Autoimmune Disease	Autoantigen	Modifications	Reference
Rheumatoid arthritis	Filaggrin	Citrullination, Carbamylation	(88)
	Fibrin	Citrullination	(89)
	Vimentin	Citrullination	(90)
	Collagen	Citrullination	(91)
	a-Enolase	Citrullination	(92)
Juvenile idiopathic arthritis	DEK protein	Acetylation	(93)
Type 1 diabetes	Insulin	Oxidation	(94)
	78-kDa Glucose-Regulated Protein (GRP78)	Citrullination	(95)
	Glutamic Acid Decarboxylase (GAD65)	Citrullination, Deamidation	(96)
	Proinsulin	Deamidation	(97)
Celiac disease	Transglutaminase	Deamidation	(87)
Systemic lupus erythematosus	SR proteins	Phosphorylation	(98)
	Histone H2B	Acetylation	(99)
	SmD1, SmD2	Methylation	(100)
	Oxidized LDL	Oxidation	(101)
	60-kDa Ro	Oxidation	(102)
Vitiligo	Tyrosinase	Oxidation	(103)
Multiple sclerosis	Myelin Basic Protein (MBP)	Citrullination	(104)

Here, we propose that some PTMs, such as citrullination, deamidation, and oxidation, may be particularly relevant with respect to autoantigen generation in AA.

### 4.1 Citrullination

Citrullination is the post-translational conversion of positively-charged arginine residues to the modified and neutrally-charged citrulline that is mediated by enzymes of the peptidyl arginine deiminase (PADI) family. Citrullination is an important post-translational modification involved in many physiological processes including skin keratinization, gene regulation, immune function, etc. (105–107). Several autoantigens harbouring citrulline residues have been identified in autoimmune diseases like RA, T1D, MS, etc. (**Table 2**). In addition to citrullinated autoantigens, autoantibodies targeting the peptidyl arginine deiminase PADI4 have also been identified in patients with RA (108). There are two ways by which citrullination may contribute immunogenicity to a self-peptide. First, side-chain citrullination can generate peptides with the capacity to bind to MHC-II alleles and thus generate a novel MHC-peptide complex for T cell activation (109). Second, citrullination may alter the T cell receptor (TCR) contact residue on the antigenic peptide, thereby potentially affecting its binding affinity. For example, the connective tissue protein vimentin has been identified as one of the potent citrullinated autoantigens in RA (90). Hill et al., demonstrated that citrullination of vimentin dramatically increases peptide binding affinity to MHC-DRB1*0401 and leads to the activation of CD4^+^ T cells in DR4-IE transgenic mice (110). Citrullinated autoantigens, such as GRP78 and GAD65, also present in T1D. Indeed, Buitinga et al., have demonstrated that the antigenicity of GRP78 is enhanced by inflammation-induced citrullination in T1D patients. This significantly increases the CD4^+^ T-cell response and is associated with high autoantibody titres (95, 111).

As one of the first differentiation proteins expressed in early anagen HF and as one of the most consistent autoantibody targets across AA in different mammalian species, we propose that citrullinated-TCHH is a promising autoantigen to investigate in AA. Indeed, peptides with citrullinated residues bind with higher affinity to HLA-DRB1*04:01, a known susceptibility locus for AA (23, 109, 112).

### 4.2 Deamidation

Deamidation is a post-translational modification catalysed by calcium-dependent TGases, which convert neutrally-charged glutamine to negatively-charged glutamic acid (113). TGases are of particular interest to the skin, because of their well-known involvement in cell differentiation and protein crosslinking processes in the skin and HF (114, 115). Tissue transglutaminase has been identified as an autoantigen in celiac disease and α-gliadin is its preferred substrate, generating novel antigenic epitopes recognized by intestinal T cells (116). Glutamine deamidation results in the generation of a more stable HLA-peptide complex, which may interact with the TCR (117). Interestingly, Arentz-Hansen et al. reported that tissue transglutaminase-mediated conversion of a single glutamine residue of α-gliadin peptide results in increased affinity of this peptide to HLA-DQ2 and is critical for T cell recognition in CD (118). In another study, it was observed that deamidation of pro-insulin, a known T1D antigen, resulted in preferential binding of the modified peptide to HLA-DQ8. Moreover, they observed a significantly higher rate of T cell activation by the modified peptide in recent-onset T1D patients (97).

TCHH is also subjected to transglutaminase-mediated deamidation, which suggests similarities with other deamidation-mediated autoimmune disorders (e.g., celiac disease). Moreover, several reports have suggested comorbidity of celiac disease in AA patients (119–121), and some have speculated that an underlying intestinal inflammation may prime patients predisposed to AA (46).

### 4.3 Oxidation

Oxidative stress can damage bio-macromolecules and is an important mediator of cytotoxicity (122). Moreover, oxidative modification of proteins elicits antibody formation in diseases such as SLE, T1D, RA, etc. (102). Antibodies have been reported in vitiligo patients to oxidized tyrosinase that are of higher affinity than those to the native form of this melanogenesis-related enzyme (103). Similarly, oxidatively-modified 60-kDa Ro, a common target of autoantibodies in SLE, appears to be more readily presented by APCs due to its altered conformation (102).

## 5 PTM Lessons for Autoantigen Discovery in AA

The HF presents particular challenges for the identification of auto-reactive targets in disorders like AA. This is because self-antigens that are expressed in the lower HF during the growth phase only (even PTM-variant forms) are not usually easily recognized by the immune system, due to the (very) low expression of MHC-I antigens in the HF epithelium. This is not the case for most other autoimmune disorders that affect normally immune-competent tissues (e.g., RA, T1D, etc.). Thus, we do not yet know whether the auto-inflammatory response to HF in AA results from a) an ectopic immune response to native (i.e., unmodified) self-antigens expressed by the healthy HF, b) a normal immune response against modified self-antigens (or neoantigens), or c) a normal immune response against self-antigens (modified/non-modified) that were not preciously visible to the immune system (because they may be conformationally-hidden or sequestered) but now become exposed and presentable in an MHC-I/II molecule-restricted manner.

Several triggers have been proposed to explain the loss of HF ‘immune privilege’ in AA, including micro-trauma, viral infection, bacterial superantigens, psycho-emotional stress, mast cell degranulation, and other immuno-genetic factors (24, 25, 123, 124). Induction of the resultant ectopic HF immune-competence, in the anagen hair bulb, may be aided by other (secondary) factors, not least peri- and/or intrafollicular secretion of IFN-γ. This potent cytokine can upregulate MHC-I expression in the epithelium of the HF anagen bulb. In that context, HF self-antigens, which are normally undetected, may now be presented to the immune system as either modified-, non-modified or neo-antigens. However, immune privilege is not ‘absolute’ in the healthy growing anagen HF. While MHC-I expression may be low in the healthy hair bulb epithelium it is not fully absent, and there are also low numbers of various immunocytes (e.g., CD4^+^ and CD8^+^ T cells, macrophages, Langerhans, mast cells) both in the HF mesenchyme (dermal sheath, dermal papilla) and even within the hair bulb itself (75). Thus, there is already some machinery that may drive or facilitate immune dysregulation in this part of the HF, which may be recruited to the earliest AA-associated changes within susceptible HFs.

A key question in the etiopathogenesis of all auto-inflammatory disorders, including AA, is what is the first strike and what is the identity of the first domino to fall in the subsequent immune-mediated cascade. Some suggestions include loss or absence of central tolerance to ectopically MHC-I-presented epitopes on HF antigens, as well as an upregulation of NKG2D ligands (e.g., MICA and ULBP) expressed in the hair bulb and associated mesenchyme (22). These initial events likely *precede* the mass infiltration of auto-reactive cytotoxic CD8^+^-NKG2D^+^ lymphocytes that wreak such destruction of the anagen hair bulb in AA.

While promiscuous expression of tissue-restricted antigens, by medullary thymic epithelial cells, is required to establish effective central tolerance (125), and loss of immune tolerance is one of the hallmarks of autoimmune diseases (126), it is not known whether intra-thymic expression of peripheral tissue-restricted antigens also extends to those proteins with PTMs. In a PTM-dependent mouse model of autoimmunity, involving the tissue-restricted self-antigen collagen type-II, T cells specific for the non-modified, i.e., native, antigen were seen to undergo efficient central tolerance. However, the PTM-variant antigen-reactive T cells escaped thymic selection, despite that the PTM-variant constituted the dominant form of the antigen in the periphery (86). This implies, at least for this case, that the PTM protein is not present in the thymus or at least is unable to induce negative selection of developing thymocytes. Thus, PTM-variant antigens may exhibit a lower level of tolerance induction than their non-PTM i.e., native parent peptide. As most self-antigens are in fact post-translationally modified, this may appear a rather curious situation. Still, it does raise the possibility that central tolerance is regulated differently for non-modified versus PTM-variants of the same self-antigen (e.g., TCHH here). Thus, T cells specific for self-antigens naturally subjected to PTM, like TCHH, may also escape effective central tolerance. Regardless, T cell reactivity to PTM-variant self-antigens is a known initiating/perpetuating factor in the progression of autoimmune diseases, as such modifications significantly affect antigen binding to MHC molecules, and consequently affect T cell activation (97, 112).

Given that the focus of the cellular and humoral immune responses to HF in AA is concentrated in/around the anagen hair bulb, it is highly likely that relevant autoantigen expression is associated with cellular proliferation and differentiation dynamics restricted to this region of the HF. Thus, likely autoantigen candidates are expressed in corresponding hair bulb cells, including melanocytes and/or proteins of early keratinocyte differentiation. *Trichohyalin* (TCHH) is a prominent example of the latter category, as it is one of the very earliest differentiation proteins to be detected in early anagen. It is of note that the *TCHH* gene is located within the so-called Epidermal Differentiation Complex (EDC), containing over 50 individual genes involved in keratinocyte differentiation (127). TCHH protein is located within granules of precursor and differentiated cells of the IRS, where it confers mechanical strength to the HF, and in precursor and differentiated keratinocytes of the hair shaft medulla (when a medulla is present). TCHH-expressing cells are first detectable in the most peripheral and proximal anagen bulb (**Figure 1**). Once these cells move distally, as they mature and differentiate, they reside in their final ‘*inner*’ root sheath position within the HF, bounded by keratinocytes of the ORS as these cells fully emerge close to the supra-bulbar HF. Thus, it is important to emphasize that proteins of earliest IRS differentiation are amongst the most ‘exposed’ in the HF to potential antigen presentation when cellular and humoral factors induce abnormal immune-competence in this tissue.

TCHH undergoes two key post-translational modifications (PTMs) during its maturation process, including citrullination by PADI enzymes and deamidation by TGases (81). Both PTMs are immunologically very important, as discussed above. Upon careful examination of co-immunostaining of anagen scalp HFs with AA patient sera and a monoclonal antibody to TCHH, we observed binding colocalization predominantly in the most exposed region of the IRS called the Henle’s layer (**Figure 2**). The latter is the first IRS sublayer to harden/cornify (128). There are a few key points worth noting from our immunohistochemical findings, which may reflect variations in the antigenicity of TCHH protein in the IRS. First, there is significant variation in the level of TCHH protein expression in the three different constituent IRS sub-layers. Second, the TCHH epitopes targeted by AA serum IgG autoantibodies appear spatially-restricted within the IRS. Third, AA patient serum IgG antibodies contain specificities that can target the earliest TCHH-positive precursor IRS keratinocytes, long before there is any discernible morphologic evidence of this HF cell layer. These TCHH-positive cells, importantly, are located most externally in the anagen hair bulb, at the interface of the proliferatively-active matrix epithelium and the mesenchymal connective tissue sheath. Thus, these precursor IRS keratinocytes can readily interact with rare surveilling immunocytes, either resident normally in this tissue or transiting from the peri-follicular vasculature. Fourth, intense AA patient serum IgG binding can also be detected circumferentially in keratinocytes located in the supra-bulbar area of the anagen HF, at a level reminiscent of IRS cells within the redox-sensitive ‘ring of fire’ (78). The latter observation suggests the potential additional involvement of oxidatively-modified autoantigens/neoantigens in AA. Thus, we propose that citrullination, deamidation, and oxidation are key PTMs involved in autoantigen generation in AA.

## 6 Conclusion and Future Prospects

The extant AA literature is voluminous, it is dominated by studies reporting the involvement of various immunocytes (including cytotoxic CD8^+^ T cells, classical natural killer (NK) cells, invariant NK T cells, γδ T cells, type-I innate lymphoid cells (ILCs) etc. (25). Remarkably, little research has focused on autoantigen discovery in AA or even on the initiating events in AA pathology. Thus, a much deeper understanding of the antigen targets of both autoantibodies and TCRs in AA will contribute towards more effective patient stratification, characterization, and management, as well as aiding the development of more targeted immunotherapies. These include the potential for induction of tolerance to the immunodominant autoantigen(s) in AA, that could provide for an ultimate cure. An important note of caution; most studies reporting on antigenic epitopes in autoimmune diseases rely on synthetic peptides or recombinant proteins (129); approaches that may ignore relevant PTMs that confer, potentially, the all-important antigenicity to these self-peptides. Thus, greater research focus needs to be directed to work on the affected tissue (e.g., scalp anagen HF) of AA patients at difference stages of their disease. Exciting recent discoveries of PTM-variant antigenic peptides in other autoimmune diseases, including RA and TID, demonstrate the very significant potential for this approach in AA.

## Author Contributions

SDJ and DJT were involved in the conceptualization, literature review, writing, and final editing of the original draft of the manuscript. Both authors have read and agreed to the published version of the manuscript.

## Funding

This work was supported by a start-up grant to DJT from University College Dublin, Ireland, and a grant to SDJ & DJT from Alopecia UK, United Kingdom [AUK2021_003].

## Conflict of Interest

The authors declare that the research was conducted in the absence of any commercial or financial relationships that could be construed as a potential conflict of interest.

## Publisher’s Note

All claims expressed in this article are solely those of the authors and do not necessarily represent those of their affiliated organizations, or those of the publisher, the editors and the reviewers. Any product that may be evaluated in this article, or claim that may be made by its manufacturer, is not guaranteed or endorsed by the publisher.
